# The Relationship Between Health Literacy and Adherence to Physical Activity Guidelines in Adults: A Scoping Review

**DOI:** 10.1177/08901171251377053

**Published:** 2025-09-06

**Authors:** Alex Lawrence, Jon Wardle, Jacqui Yoxall

**Affiliations:** 1 Faculty of Health, 4571 Southern Cross University, Coffs Harbour, NSW, Australia; 2 National Centre for Naturopathic Medicine, Faculty of Health, 4571 Southern Cross University, Gold Coast, QLD, Australia

**Keywords:** health literacy, physical activity, health promotion, health numeracy

## Abstract

**Objective:**

This scoping review aimed to synthesise research on the relationships between health literacy and adherence to physical activity guidelines in adults.

**Data Source:**

A search of MEDLINE, ProQuest, Scopus, CINAHL, Web of Science (Core Collection), PubMed, and PsycINFO was conducted using keywords. Observational and intervention studies written in English were reviewed.

**Inclusion and Exclusion Criteria:**

Eligible research studies used a validated, objective measure of health literacy. Physical activity needed to be reported as either a primary or secondary outcome, and groups needed to be dichotomised as physically active, inactive, or similar.

**Extraction:**

Out of 2098 articles identified, 19 met the inclusion criteria.

**Synthesis:**

A numerical analysis of the studies was performed. A narrative summary supplemented the analysis to synthesise the main themes and patterns.

**Results:**

Fifteen studies examined the association between total health literacy scores and achieving >150 minutes of moderate-to-vigorous physical activity weekly. Nine studies reported a positive association, while others found no significant association. In particular, studies using self-reported physical activity more frequently found an association, whereas no association was found when using objective physical activity measures.

**Conclusion:**

The findings of this review were inconclusive. The lack of a standardised health literacy instrument presents a barrier to progress in the field of physical activity and health literacy research. Moreover, longitudinal relationships between health literacy, mediators and physical activity must be investigated.

## Objective

Health literacy (HL) refers to an individual’s capacity to navigate, understand, and use health-related information to manage their health.^1,2^ Low health literacy is linked to poor health outcomes, including higher mortality rates, medication non-adherence, and difficulties navigating health systems,^3-5^ and increased risk of unhealthy behaviours, such as physical inactivity.^5^ Such associations make HL particularly important for public health and health promotion efforts.

Approximately one-third (31%) of the global adult population, or 1.8 billion people, are physically inactive.^6^ Physical inactivity is a major modifiable risk factor of all-cause mortality, accounting for 6% of premature deaths worldwide.^7,8^ Reducing physical inactivity by 10% or 25% could prevent more than 533 000 and over 1.3 million deaths, respectively, each year.^8^ Increasing physical activity (PA) prevents, mitigates, and ameliorates the effects of many non-communicable diseases, injuries, and associated comorbidities.^9^ Furthermore, achieving the recommended levels of PA can reduce the risk of all-cause mortality by 75%.^10^ While meeting the PA guidelines is attainable for most individuals, adopting and maintaining an active lifestyle also requires a combination of cognitive, behavioural, and self-regulatory skills to overcome motivational and scheduling barriers.^11^ As efforts to achieve global PA targets continue, the nexus between HL and adherence to PA guidelines is becoming increasingly relevant.

Previous systematic reviews have concluded that there is a positive relationship between HL and PA levels across different age groups.^12,13^ However, these findings referred to an increase in PA irrespective of whether such an increase meets recommended PA guidelines. Although any increase in PA is broadly beneficial, typically, the interpretation of population surveillance data and the implementation of public health policy often centres on whether recommended guidelines for PA are met. By focusing on PA guidelines, the design of this study aligns with public health priorities; targeting the relationship between HL and the threshold of PA most likely to result in meaningful health improvements.

The variation in PA and HL measurement tools and the complex nature of the HL construct generally make comparing and synthesising findings across studies challenging.^14,15^ Scoping reviews, however, are particularly valuable in such cases, as they are well-suited to synthesise heterogeneous literature.^16^ To our knowledge, no scoping review on HL and adherence to PA guidelines has been published.

This scoping review aims to identify and synthesise research on the relationships between HL and adherence to PA guidelines. Specifically, the proposed review seeks to answer the following review questions: (i) What is the association between HL and adhering to PA guidelines in the general adult population?; (ii) What HL constructs are being measured, and what instruments are used to assess them?; (iii) How does implementing HL interventions compare to no intervention or standard care impact adherence to PA guidelines in the general adult population?; and (iv) What are the patient and social-level factors suggested to influence the connection between HL and health numeracy, and adhering to PA guidelines?

## Method

This scoping review followed the Joana Briggs Institute (JBI) approach to scoping reviews.^17^ The JBI approach was used for its methodological quality and the availability of tools to guide scoping reviews.^18^ The research team developed the review protocol and registered with the Open Science Framework on the 20th of February 2024 (https://osf.io/smqfj). This report was written using the Preferred Reporting Items for Systematic Reviews and Meta-Analyses Extension for Scoping Reviews (PRISMA-ScR) checklist.^19^ To identify the key concepts relevant to the primary research question, “What is the association between health literacy and physical activity in the general adult population?” we used the Population, Concept, and Context (PCC) framework recommended by the Joanna Briggs Institute.

### Eligibility Criteria

The review included observational or intervention studies written in English and involved the general adult population (18 years or older). The English language restriction was due to resource constraints. Studies with adults and children were considered if they reported data separately for adults. However, studies focused exclusively on adolescents or children were excluded.

Eligible research studies used a validated, objective measure of HL. Studies that relied upon self-assessed measures, where participants estimate their HL levels, were excluded as these assessments are generally intended for use in clinical settings rather than research purposes. For intervention studies, HL needed to be objectively measured both at baseline and after the intervention and show improvements to be included.^20^

To qualify for inclusion, PA needed to be reported as either a primary or secondary outcome, and groups needed to be dichotomised as physically active or inactive or similar. Self-reported measures of PA were accepted due to their widespread use in large-scale observational studies and the application of standardised thresholds (eg, ≥150 minutes of PA per week). For this study, people who do not meet recommended levels of regular PA were defined as “physically inactive”. Where pedometers were used to measure PA, participants must achieve at least 7000 steps per day to be considered sufficiently active.

### Information Sources

The search strategy followed the Peer Review of Electronic Search Strategies standard.^21^ An initial search on PubMed was performed to identify relevant articles based on keywords in titles, abstracts, and index terms. Subsequently, a Health Sciences Librarian assessed the strategy to balance breadth, comprehensiveness, and feasibility. A search strategy was then tested on PubMed to verify and refine the search query as needed to ensure it captured a significant number of relevant studies. Once finalised, the strategy was adjusted to suit other databases’ syntax and subject headings. Validations and updates continued throughout the review process to maintain the effectiveness of the PubMed search strategy. The following bibliographic databases were searched: MEDLINE, ProQuest, Scopus, CINAHL, Web of Science (Core Collection), PubMed, and PsycINFO. The search was unrestricted by date. To achieve literature saturation, we examined the reference lists of included studies and relevant reviews identified during the search process.

### Selection of Sources of Evidence

**Figure 1.**
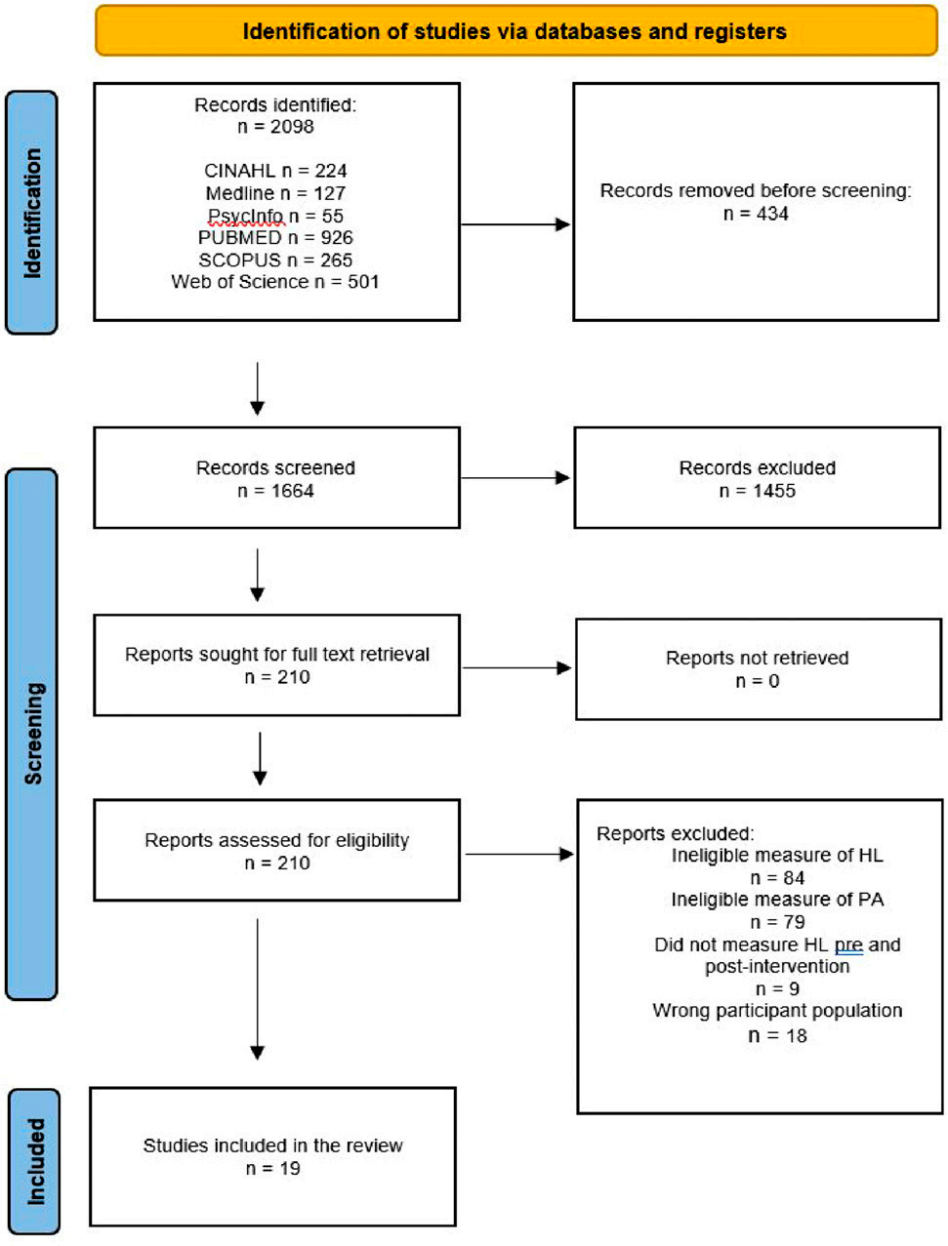
Flow Diagram of the Article Selection Process according to the PRISMA-ScR

The results of each review were exported to EndNote 20 (Clarivate Analytics, PA, USA) to remove duplicates. The remaining articles were imported into Covidence (https://www.covidence.org/) for further duplicate detection. One reviewer screened the titles and abstracts; potentially relevant articles were retrieved in full and added to Covidence. Two independent reviewers assessed the full texts against the inclusion criteria, with any exclusions documented according to JBI guidelines. Discrepancies were resolved through discussion or by consulting an additional reviewer. The search results and study inclusion process are presented using the PRISMA-ScR flow diagram (Figure 1).

### Data Extraction

The Joanna Briggs Institute standardised data extraction form guided data extraction from each included study. The data extraction form was piloted on a small subset of studies to ensure clarity, completeness, and consistency in data collection. The form was refined based on issues identified during this pilot phase. Data extraction was performed independently by one reviewer (AL). The reviewers were not required to contact study authors or investigators to clarify any ambiguous or missing data. The reason for exclusion has been noted and described in the PRISMA flow diagram (Figure 1). Consistent with guidance on scoping reviews, no methodological quality appraisal of the included studies was performed.

### Data Analysis

A numerical analysis of the nature and extent of the studies was performed. A narrative summary supplemented the analysis to synthesise the main themes, patterns, and conclusions from the review, explaining how the results align with the review’s aim and questions.

## Results

The selection of sources of evidence is illustrated in Figure 1. A total of 2098 articles were identified from a search of electronic databases. After removing 434 duplicates, 1664 titles and abstracts were screened. Full texts were retrieved for 210 studies, of which 191 were excluded for reasons such as not adequately measuring HL (n = 84), did not measure HL pre- and post-intervention (n = 10), including the wrong participant population (n = 18), and issues with validity, alignment with guidelines, and classification of PA (n = 79). A total of 19 articles met the inclusion criteria and were included in the review.

### Study Characteristics

The study characteristics are summarised in Table 1 (Additional file 1). Among the studies selected for inclusion, most were cross-sectional design (n = 13),^22-34^ followed by longitudinal design (n = 3),^35-37^ prospective cohort design (n = 1),^38^ correlational study (n = 1),^39^ a combined cohort and case-control methods (n = 1).^40^ Most studies were published in Europe (n = 10),^25,27,29,30,34-38,40^ including Germany (n = 2),^29,36^ Denmark (n = 2),^30,38^ Netherlands (n = 2),^35,40^ United Kingdom (n = 1),^37^ Italy (n = 1),^34^ Ireland (n = 1),^25^ and a multi-national European study (n = 1).^27^ Otherwise, studies were published in Australia (n = 2),^26,28^ Turkey (n = 2),^24,39^ and there was 1 study each from, Canada,^22^ Iran,^33^ Japan,^31^ Singapore,^23^ and the United States of America.^32^

**Table 1.** Summary of Study Designs, Participants, and Intervention Procedures

No	Authors, Year of Publication, Country	Research question	Study design Population	Health literacy Scale Health literacy domains being measured	Measure of PA	Results
1	Ahcioglu and Yilmazel, 2022, Turkey	To examine the health literacy and associated factors among coronary artery disease patients	Correlational study 275 hospitalised patients (≥50 years) diagnosed as any CVD in the cardiology service and coronary intensive care unit	Turkish Health Literacy Scale-32 (TSOY-32) Not mentioned	Self-reported measure of PA with data being gathered via face-to-face interviews	51.5% of participants with inadequate HL were sufficiently PA (≥30 min/d), compared to 48% participants with inadequate HL who were insufficiently PA (<30 min/d). The results were significant (χ^2^ = 7.5000, *P* < 0.05)
2	Al Sayah et al 2016, Canada	To investigate whether inadequate health literacy is associated with self-reported moderate to vigorous physical activity in community-dwelling adults	Cross sectional study 1296 participants	Three validated questions. (1) problems learning; (2) confidence filling out medical forms; (3) Help reading	Self-reported measure of PA using the lesure score index of the goldin leasure-time exercise questionnaire to estimate physical activity Objective measure using pedometer	Crude model: A significant association was found between participants with adequate HL and likelihood to achieve sufficient PA as measured by ≥ 150 mins/wk (OR 0.51, 95% CI 0.34-0.77, *P* = 0.001); and ≥600 MET/wk (OR 0.53, 95% CI 0.34-0.80, *P* = 0.003) No association was found between participants with adequate HL and likelihood to achieve sufficient PA (≥8000 steps/d) (OR 1.02, 95% CI 0.59-0.1.77, *P* = 0.94) Adjusted model*: A significant association was found between likelihood to achieve sufficient PA (≥150 mins/wk) and adequate HL (OR_adj_ 0.63, 95% CI 0.41-0.97, *P* = 0.037) A non-significant association was found between likelihood to achieve sufficient PA (≥600 MET/wk) and adequate HL (OR_adj_ 0.65, 95% CI 0.42-1.01, *P* = 0.06) No association was found between likelihood to achieve sufficient PA (≥8000 steps/d) and adequate HL (OR_adj_ 1.27, 95% CI 0.70-2.29, *P* = 0.43) *Adjusted for age, gender, education, income, employment, ethnicity, smoking status, BMI, and number of chronic illnesses
3	Asharani et al 2021, Singapore	To examine the functional health literacy of the nation, factors associated with health literacy, and its relationship with diabetes recognition	Cross sectional study 2895 participants	The brief health literacy screen (1) Problems learning (2) Confidence filling out medical forms (3) Help reading	Self-reported measure of PA using the Global Physical Activity Questionnaire (GPAQ)	84.4 % of participants with adequate HL were sufficiently PA (≥150 mins/wk), compared to 79.3% of participants with inadequate HL who were insufficiently PA (<150 mins/wk). The results were not significant (*P* = 0.06) Adjusted model*: No association was found between likelihood to achieve sufficient PA (≥150 mins/wk) and adequate HL (OR 1.1, 95% CI 0.7-1.6, *P* = 0.78) *Sociodemographic variables
4	Brors et al 2022, Denmark	To investigate the associate between health literacy, behavioural and psychologic risk factors, BMI, physical activity, anxiety depression, and smoking status in patients treated with percutaneous coronary intervention, as well as to assess changes in these factors over a 6-month follow-up period	Prospective cohort study 3417 patients aged 10 years and over	Four of the 9 measurements in the Health Literacy Questionnaire (HLQ) (1) Access (2) Understanding (3) Appraise (4) Social	Self-reported measure	Crude model: A significant association was found between participants adherence to PA recommendation (≥150 mins/wk) and adequate HL: *social support* (coef. 22.81, 95% CI 15.24-30.39, *P* < 0.001); *appraisal* (coef. 14.22, 95% CI 8.03-20.40, *P* < 0.001); *access* (coef. 21.73, 95% CI 16.42-27.04, *P* < 0.001); and *understanding* (coef. 26.04, 95% CI 19.80-32.28, *P* < 0.001) Adjusted*: A significant association was found between participants adherence to PA recommendation (≥150 mins/wk) and adequate HL: *social support* (coef. 20.55, 95% CI 12.90-28.19, *P* < 0.001); *appraisal* (coef. 11.80, 95% CI 5.58-18.02, *P* < 0.001); *access* (coef. 17.10, 95% CI 11.35-22.85, *P* < 0.001); and *understanding* (coef. 20.51, 95% CI 13.89-27.14, *P* < 0.001)
5	Emiral et al 2021, Turkey	To asses the potential determinates for both knowledge level of metabolic syndrome and health literacy level among the adult population in Western Turkey	Cross sectional study 774 participants	Health Literacy Scale European Union (HLS-EU-Q16) (1) Health care (2) Disease prevention (3) Health promotion	Self-reported measure of PA using a single question	Participants with higher mean HL score (33.40±8.50 vs 31.82±7.02) had a higher prevalence of sufficient PA (≥30 min/d). The results were significant (*P* = 0.011)
6	Goboers et al 2014, Netherlands	To assess the association of health literacy and physical activity and nutritional behavior in older adults, and to determine if this association is mediated by social cognitive factors	Longitudinal study 525 community-dwelling older adults (≥55 years)	Three validated questions (1) Problems learning (2) Confidence filling out medical forms (3) Help reading	Self-reported measure of PA using the SQUASH questionnaire	32.4% of participants with inadequate HL were insufficiently PA (<150 mins/wk), compared to 21.7% of participants with adequate HL who were insufficiently PA (<150 mins/wk). The results were significant (*P* = 0.007) Crude model: A significant association was found between insufficiently PA (<150 mins/wk) and inadequate HL (OR 1.74, 95% CI 1.16-2.95, *P* = 0.007). Adjusted model*: A significant association was found between insufficiently PA (<150 mins/wk) and inadequate HL (OR_adj_ = 1.52, 95% CI 1.00-2.31, *P* = 0.020) Self-efficacy mediated 32% of the association between HL and sufficient PA. Neutral/negative attitude and low risk perception failed mediate association * Adjusted for condition, age and gender
7	Goboers et al 2016, Netherlands	To assess the associations between health literacy and various health behaviours and social factors among older adults, as well as to investigate whether social factors moderate the associations	Cohort and case control study. 3241 older adults (≥65 years)	Three validated questions (1) Problems learning (2) Confidence filling out medical forms (3) Help reading	Self-reported measure of PA using a single question	33.5% of participants with inadequate HL were insufficiently PA (<150 mins/wk), compared to 27.6% of participants with adequate HL who were insufficiently PA (<150 mins/wk). The results were significant (*P* < 0.01) Crude: A significant association was found between insufficiently PA (<150 mins/wk) and inadequate (OR 1.32, 95% I 1.13-1.55, *P* < 0.01) Adjusted*: A significant association was found between insufficiently PA (<150 mins/wk) and inadequate HL (OR_adj_ = 1.31, 95% CI 1.11 – 1.54, *P* < 0.001) Social factors (loneliness, social support, social activities social contacts, and living situation) failed to mediate association * adjusted for age and gender
8	Gibney and Doyle 2017, Ireland	To investigate the relationship between self-rated health literacy and exercise frequency among individuals aged 50+ in Ireland	Cross sectional study 389 adults aged 50+	HLS-EU-Q47 (1) Health care (2) Disease prevention (3) Health promotion	Self-reported measure of PA using a single question	Participants with lower mean HL sub-domain health promotion score (32.0±10.3) had a higher prevalence of insufficient PA (<150 mins/wk), compared with participants with higher mean HL sub-domain health promotion score (36.2±8.3) had a higher prevalence of sufficient PA (≥150 mins/wk). The results were significant (*P* = 0.010) Participants with lower mean score for HL sub-domain health promotion: Access (6.1.0±2.3) had a higher prevalence of insufficient PA (<150 mins/wk), compared with participants with higher mean HL sub-domain health promotion: Access (7.0±1.8) had a higher prevalence of sufficient PA (≥150 mins/wk). The results were significant (*P* = 0.004) Adjusted*:A significant association was found between sufficient PA (≥150 mins/wk) and higher HL domains: Disease prevention-understanding (OR_adj_ = 1.18, 95% CI 1.03 – 1..34, *P* < 0.05); health promotion-access (OR_adj_ = 1.13, 95% CI 1.00 – 1..29, *P* < 0.05); health promotion-understanding (OR_adj_ = 1.15, 95% CI 1.02 – 1..32, *P* < 0.05); and, health promotion-evaluation (OR_adj_ = 1.13, 95% CI 1.01 – 1..26, *P* < 0.05) The non-significant associations and non-associations between sufficient PA (≥150 mins/wk) and the other HL indices were not reported *Adjusted for age, gender, employment status, marital status, education, financial deprivation, social status and physical limiting illness
9	Jayasinghe et al 2016, Australia	To investigate the impact of health literacy on Health-Related Quality of Life (HRQoL)	Cross sectional study 379 participants	Health Literacy Management Scale (HeLMS) (1) Attitudes (2) Understand (3) Social support (4) Socioeconomic considerations (5) Assess (6) Interactive (7) Being proactive (8) Apply	Self-reported measures	62.7% of participants with inadequate HL were insufficiently PA (<150 mins/wk), compared to 48% participants with adequate HL who were insufficiently PA (<150 mins/wk). The results were significant (*P* < 0.001)
10	Jennings et al 2013, Multi-national (Europe)	To explore the association between illness perceptions and health literacy with sociodemographic characteristics and risk factors, health-related quality of life, anxiety and depression	Cross sectional study 3408 participants (aged 18-80 years) with heart conditions	Only the 9^th^ scale of the Health Literacy Questionnaire (HLQ-9) was used (1) Understanding health information	Self-reported measures	Participants with lower HL levels were significantly associated with insufficient PA (*P* < 0.001)
11	Joshi et al 2014, Australia	To compare primary car patients with and without sufficient health literacy in terms of their lifestyle risk factors and explore associated with receiving advice and referral for the risk factors from their GP.	Cross sectional study 739 participants	HeLMS (1) Attitudes (2) Understand (3) Social support (4) Socioeconomic considerations (5) Assess (6) Interactive (7) Being proactive	Self-reported measures	A significant association was found between insufficient PA (<150 mins/wk) and participants with inadequate HL (OR_adj_ = 1.81, 95% CI 1.34 – 2.43, *P* < 0.001)
12	Jürgensen et al 2024, Germany	To explore and describe the health status, health-related behavior, and health literacy levels of first-year health professional students, as well as to investigate potential differences between 2 groups of students	Cross sectional study 90 first year health professional students	HLS-EU-Q16 (1) Health care (2) Disease prevention (3) Health promotion	Self-reported measures	65.1% of participants with inadequate HL were insufficiently PA (<2h/wk), compared to 61.5% participants with adequate HL who were insufficiently PA (<2h/wk). The results were not significant (*P* = 0.831)
13	Juul et al 2018, Denmark	To investigate associations between health literacy and diet and physical activity, and motivation and diet and physical activity in Danish people with type 2 diabetes	Cross sectional study 194 adults with type 2 diabetes	HLS-EU-Q16 (1) Functional (2) Critical (3) Communicative	Self-reported measures using the SDSCA.	Crude: A non-significant association was found between frequency of achieving sufficient PA (≥30min/d) and functional HL (β 0.31, 95% CI −0.12-0.74), and communicative HL (β 0.38, 95% CI −0.16-0.92) No association was found between frequency of achieving sufficient PA (≥30min/d) and total HL (β 0.06, 95% CI −0.12-0.74), and critical HL (≥30min/d) (β 0.17, 95% CI −0.33-0.67) Adjusted: No association was found between total HL, functional HL, communicative HL or critical HL and frequency of achieving sufficient PA (≥30min/d) *Adjusted for age, gender education level, diabetes duration, and other HL scores
14	Kobayashi et al 2016, United Kingdom	To investigate the relationship between health literacy and participation in weekly moderate to vigorous physical activity over an 8-year period among older adults	Longitudinal study 4345 adults aged 52-79 from the English longitudinal study	Validated four-item measure from the organisation for Economic Co-operation and development internation adult literacy survey (1) Reading comprehension	Self-reported measures	Crude:An association was found between sufficient PA (≥150 mins/wk)and participants with medium HL (OR 1.83, 95% CI 1.41-2.37); and high HL (OR 2.83, 95% CI 2.25-3.57) Adjusted*:An association was found sufficient PA (≥150 mins/wk) and participants with medium HL (OR 1.29, 95% CI 0.95-1.75), and high HL (<150 mins/wk) (OR 1.53, 95% CI 1.16-2.01) Adjusted**: An association was found between sufficient PA (≥150 mins/wk) and participants with medium HL (OR 1.21, 95% CI 0.89-1.64), and high HL (OR 1.37, 95% CI 1.04-1.81) *Adjusted for age, sex, education attainment, net non-person wealth, self-rated health, limiting long-standing illness, and IADL limitation compliance **Additional adjustments for memory an verbal fluency
15	Koch et al 2023, Germany	To monitor the health of trainees in different sectors over time, ending during the COVID-19 pandemic, and to examine the association between health literacy and health/health behaviour	Longitudinal study 129 trainees from various sectors	HLS-EU-Q16 (1) Health care (2) Disease prevention (3) Health promotion	Self-reported measures	No association was found between HL (at baseline) and PA (3 months) (*P* = 0.124)
16	Nagaki et al 2023, Japan	To evaluate the association between physical activity levels and different health literacy domains in patients with Parkinsons disease	Cross sectional study 141 Japanese patients with Parkinsons disease aged 18 years or older	Functional, Communicative, ad Critical Health Literacy (FCCHL) scale (1) Functional (2) Critical (3) Communicative	Self-reported measures using the IPAQ short form	Crude model: A significant association was found between participants adherence to PA recommendation (≥150 mins/wk) and critical HL (OR 2.12, 95% CI 1.06-4.22, *P* = 0.03) A non-significant association was found between participants adherence to PA recommendation (≥150 mins/wk) and functional HL (OR 1.40, 95% CI 0.77-2.53, *P* = 0.26) and communicative HL (OR 1.42, 95% CI 0.65-3.11, *P* = 0.38) Adjudged model^*^: A significant association was found between participants adherence to PA recommendation (≥150 mins/wk) and critical HL (OR_adj_ 2.46, 95% CI 1.16-5.19, *P* = 0.02) A non-significant association was found between participants adherence to PA recommendation (≥150 mins/wk) and functional HL (OR_adj_ 1.58, 95% CI 0.84-2.96, *P* = 0.16). And communicative HL (OR_adj_ 1.51, 95% CI 0.66-5.19, *P* = 0.38) * adjusted for age, gender, PD duration, and academic background
17	Reider et al 2017, United States of America	To determine whether health literacy was associated with meeting the recommended guidelines for physical activity in people with multiple sclerosis	Cross sectional study 9019 adults with multiple sclerosis	METER (1) Functional health literacy	Self-reported measures using the Health Information National Trends (HINTS) survey	No association was found between adherence to PA recommendation (≥150 mins/wk) and functional HL (OR 0.91, 95% CI 0.78-1.06, *P* > 0.05)
18	Shahavandi et al 2021, Iran	To evaluate the associations between health literacy and healthy eating index (HEI) in adults	Cross-sectional study 261 Iranian adults aged 18 to 65 years	Health Literacy for Iranian Adults (HELIA) questionnaire (1) Accessing (2) Reading (3) Comprehension (4) Assessment (5) Decision-making	Self-reported measures using the IPAQ short form	A significant association was found between adherence to PA recommendation (≥150 mins/wk) and HL (β 0.12, 95% CI -0.05-1.72, *P* = 0.03)
19	Zanobini et al 2021, Italy	To evaluate health literacy levels and their associations with sociodemographic factors and health-related behaviours in a representative sample of the Tuscan population	Cross-sectional study 7151 adults	The Italian version of the HLS-EU-Q6 (1) Health care (2) Disease prevention (3) Health promotion	Self-reported measures	A significant association was found between adherence to PA recommendation (≥150 mins/wk) and HL (*P* < 0.001) Adjudged model^*^: A significant association was found between participants adherence to PA recommendation (≥150 mins/wk) and HL (OR_adj_ 0.60, 95% CI 0.50-0.73, *P* = 0.000) *Adjusted for age, sex, educational level, occupation status, financial status and nationality

### Health Literacy

Various HL assessment tools were used in the included studies. Of the 19 studies, 3 used only a portion of a survey.^27,37,38^ Most studies used a version of the European health literacy survey (HLS-EU), including the HLS-EU-Q16 (n = 4),^24,29,30,36^ HLS-EU-Q47 (n = 1),^25^ and the Italian version of the HLS-EU-Q6 (n = 1),^34^ The Brief Health Literacy Screen was utilised in 4 studies.^22,23,35,40^ Two studies used the Health Literacy Questionnaire (HLQ-9); however, neither study used the entire scale. Specifically, Brors, Dalen^38^ used 4 of the 9 measurements: accessing, understanding, and appraising health information and the availability of social support to assist in managing health. Jennings, Astin 27 only measured understanding of health information. The Health Literacy Management Scale was used in 2 studies.^26,28^ The Medical Term Recognition Test,^32^ the Turkish Health Literacy Scale-32,^39^ the Newest Vital Sign,^39^ the Organisation for Economic Co-operation and Development International Adult Literacy Survey,^37^ the Health Literacy for Iranian Adults tool,^33^ and the Functional, Communicative, and Critical Health Literacy Scale^31^ featured in 1 study each.

### Physical Activity

Eighteen studies relied on self-reported measurements of PA. Of these, 12 used a direct question,^24-29,34,36-40^ 1 used the Global Physical Activity Questionnaire,^23^ 1 used the SQUASH questionnaire,^35^ 1 used the SDSCA questionnaire,^30^ and 2 employed the IPAQ short form.^31,33^ Additionally, 1 study utilised the Health Information National Trends Survey.^32^ One study combined both an objective measure, using a pedometer, with a self-reported measure of PA, specifically the Leisure Score Index from the Godin Leisure-Time Exercise Questionnaire.^22^

### Findings

Fifteen studies examined the association between total HL scores and PA of ≥150 minutes of MVPA per week. Nine studies found a significant positive association between PA and HL,^22,24,26-28,33-35,40^ while 4 studies found no such association.^23,29,30,32^ One study reported a significant association using unadjusted odds ratios, but this association became non-significant after adjusting for covariates.^37^ Another study found that individuals with inadequate HL were more likely to engage in sufficient PA.^39^

One study specifically explored the relationship between PA of ≥600 MET and HL.^23^ The unadjusted model indicated a significant association, but this association was no longer significant after adjustment for covariates. No association was found between adequate HL and the likelihood of achieving sufficient PA (≥8000 steps/day) in either crude or adjusted models.

Five studies investigated the relationship between PA of ≥150 minutes per week and a specific subdomain of HL. One study consistently found significant associations between PA and the subdomains of social support, appraisal, access, and understanding in both crude and adjusted models.^33^ Communicative HL was found to have a non-significant association with PA in 2 studies using unadjusted models; however, both studies reported no association when using adjusted models.^30,31^ Three studies examined functional HL. Two reported non-significant associations in crude models^30,31^ and 1 found no association.^32^ Only 2 of these studies used adjusted models, and both reported no association.^30,31^ One study focused on 3 HL domains: Disease Prevention, Health Promotion, and Healthcare, each with 4 sub-indices: Accessing, Understanding, Evaluating, and Applying.^25^ Significant findings were reported only for Health Promotion (combined), Disease Prevention: Understanding, Health Promotion: Accessing, Health Promotion: Understanding, and Health Promotion: Evaluating.^25^

No studies reported on the direct relationship between health numeracy (HN) and adherence to PA guidelines. One study investigated if self-efficacy, neutral/negative attitude and low-risk perception mediated the relationship between HL and sufficient PA.^35^ Self-efficacy mediated 32% of the association between HL and sufficient PA, while neutral/negative attitude and low-risk perception did not show as mediators.^35^

## Discussion

### Summary of Key Findings

This scoping review aimed to identify and synthesise existing research on the relationship between HL and PA. This review builds on previous work,^12,13^ explicitly focusing on HL and its relationship with adherence to PA recommendations. However, the findings of this scoping review are inconsistent with previous work.^12,13^ This discrepancy may be attributed to differing inclusion criteria or publication timelines. Three studies not documented by Buja^12^ were identified in this review.^25,26,32^ Considering that this search, which has a narrow focus on PA guidelines, identified missed studies, it is plausible that previous evaluations may have overlooked further research outside this review’s scope. Furthermore, half of the studies included in this review were published after Buja.^12^ Considering the concept of HL is changing and increasing in complexity, more recent studies might indicate a shift in the relationship between HL and PA. Therefore, this review may provide a new viewpoint on the impact of HL on PA, reflecting the changing nature of HL itself. Nevertheless, the changing concept of HL is improbable in explaining the disparity in our results, as previous studies also documented substantial variation in the measure of HL between studies.^12,13^

The most likely explanation for the variation in results is in the measurement of the PA. This study specifically reviewed adherence to PA guidelines, while other studies included a broader range of PA measures, including PA level (low, moderate, high), mean PA, and PA intensity (light, moderate, vigorous). PA guidelines are intended to be easy to understand. Compared to self-directed PA, the relative simplicity of following set guidelines may help explain differences observed in the findings.

The results of our review suggest that there is some evidence to support an association between HL and PA. Compared with individuals with low HL, people with adequate HL were more likely to comply with PA recommendations and maintain a more active lifestyle. Nevertheless, all of these studies depend on self-reported PA data, which is susceptible to recall bias and inaccuracies in documenting actual PA.^41^ This implies the necessity of exercising caution when interpreting the results and restricts the reliability of the findings. Therefore, although some evidence suggests an association between HL and PA, the dependence on self-reported data introduces constraints that must be resolved in subsequent research.

Interestingly, the only study that used an objective measure of PA reported no association between HL and PA.^22^ This is a key finding that contrasts with studies relying on self-reported data. An explanation for this may be the role of self-perception in HL. Individuals with higher HL might report PA levels that align more with health recommendations, even if their actual activity levels do not differ significantly from individuals with lower HL when measured objectively. Another explanation is HL may influence how PA is reported in self-report questionnaires. Participants with inadequate HL might have underreported their actual PA compared to those with adequate HL, potentially due to misunderstanding or misinterpreting the questions. Overall, the evidence on the relationship between HL and PA remains inconclusive. While there is some indication of a link, the current body of research is insufficient to draw any conclusions.

### Gaps in the Literature and Future Recommendations

Most studies are cross-sectional, limiting conclusions about causality between HL and PA. Longitudinal or intervention studies would provide stronger evidence for causation. Future studies should prioritise longitudinal studies to progress knowledge of the interaction between HL and adherence to PA guidelines. Moreover, none of the intervention studies satisfied the inclusion requirements, indicating a gap in the research that should be addressed.

Half of the studies in our review used multivariate logistic regression analysis. This approach enhanced the clarity of the results by accounting for various confounders and allowed for the measurement of odds ratios, which quantifies the strength of the association between each predictor and the outcome, making the results easier to interpret.^42^ However, the selection of confounding variables varied across studies, which complicated synthesising the overall findings. Furthermore, some studies controlled for factors that may lie within the causal pathway. Factors like knowledge and income might mediate the relationship between HL and health outcomes. This “overadjustment” could have underestimated the true effect of HL by masking its direct influence on PA.

Only 1 study in this review actually investigated possible mediating effects, identifying self-efficacy as mediating the relationship between HL and PA. This finding is consistent with previous empirical studies that found that self-efficacy mediates HL and self-care behaviours.^43^

HN has often been overshadowed in broader discussions of HL, either being considered a subset of HL or ignored entirely. Defined as *“the degree to which individuals have the capacity to access, process, interpret, communicate, and act on numerical, quantitative, graphical, biostatistical, and probabilistic health information needed to make effective health decisions”*,^44^ HN might be particularly relevant to PA behaviours. For instance, setting goals, such as achieving 10 000 steps daily or following public health guidelines on PA, are mainly numerical. Without adequate HN, individuals may struggle to effectively manage and maintain appropriate levels of PA. Future research exploring the relationship between HN and adherence to PA guidelines is needed, as it may offer valuable insights into how numeracy skills influence PA behaviours and inform more effective health promotion strategies.

This gap in the evidence base is particularly striking given the global policy emphasis on health literacy. There is a critical need for pragmatic, well-funded research, particularly real-world evaluation studies, to assess whether and how HL and HN interventions translate into measurable improvements in population-level PA.

### Strengths and Limitations

Our study has several strengths. First, the extensive search strategy makes it unlikely that any significant studies were missed. Additionally, the low risk of bias from selective reporting strengthens the study’s objectivity, as many published studies show no relationship between HL and compliance with PA recommendations. However, there are some limitations. This review did not assess the methodological quality of the selected studies, which restricts our ability to assess the overall findings. Furthermore, including only English-language articles may have excluded important studies published in other languages, potentially introducing a language bias and limiting the generalisability of the results.

## Conclusion

To our knowledge, this is the first scoping review exploring the association between HL, HN and adherence to PA guidelines in adults. Despite the growing prevalence and importance of HL research, this scoping review reports substantial heterogeneity in the measure of HL, making it difficult to confidently conclude that HL is associated with adherence to PA guidelines in adults. This finding is particularly striking given that HL has become a dominant feature of healthy public policy. Future studies should prioritise the inclusion of HN, adopt longitudinal designs, standardise HL measures, and explore potential mediators such as self-efficacy through intervention-based research.

**So what?**

**Implications for Health Promotion Practitioners and Research**

The findings from this review reinforce the need for further research to better understand the association between HL and PA. The review’s inconclusive results suggest that there is not yet a consistent link between HL and compliance with PA guidelines. This raises concerns about assuming that improving HL alone will significantly enhance PA. Although this review was restricted to adherence to PA guidelines, the mixed results presented in this review may also extend to other areas of behaviour, such as diet adherence and lifestyle change. Policymakers should therefore be cautious when relying on HL as the primary driver of interventions aimed at raising population PA levels to meet guidelines. In practice, healthcare professionals should continue to acknowledge HL in the provision of services. However, when implementing strategies to improve PA adherence, focusing on approaches with a stronger evidence base, such as goal setting, might offer a more reliable solution.

## Supplemental Material

Supplemental Material - The Relationship Between Health Literacy and Adherence to Physical Activity Guidelines in Adults: A Scoping Review Supplemental Material for The Relationship Between Health Literacy and Adherence to Physical Activity Guidelines in Adults: A Scoping Review by Alex Lawrence, Jon Wardle PhD, and Jacqui Susan Yoxall in American Journal of Health Promotion

## Data Availability

The datasets used and/or analysed during the current study are available from the corresponding author on reasonable request.*
