# Supplementary material for: The Relationship Between Health Literacy and Adherence to Physical Activity Guidelines in Adults: A Scoping Review
Source: Am J Health Promot. 2025 Sep 6;40(3):378–91. doi: 10.1177/08901171251377053 (PMC12861545; doi:10.1177/08901171251377053)
Supplement: Supplemental Material - The Relationship Between Health Literacy and Adherence to Physical Activity Guidelines in Adults: A Scoping Review [file sj-pdf-1-ahp-10.1177_08901171251377053.pdf]

Search strategy

Database: **Pubmed**
Search date: 13/02/2024

	**Search strategy**	**Results**
#1	Exercise[mh] OR "Sedentary Behavior"[mh]	258,544
#2	"Physical activ*"[tiab] OR "Physical inactiv*"[tiab] OR Exercis*[tiab] OR "Sedentary Behav*"[tiab]	501,630
#3	#1 OR #2	592,615
#4	“Health Literacy”[mh]	9,863
#5	“Health numeracy"[tiab] OR “Health Knowledge”[tiab] OR “Health Literacy”[tiab]	15,896
#6	#4 OR #5	25,346
#7	#3 AND #6	1,159
#8	"Digital literacy" [Tiab] OR eliteracy[Tiab] OR "physical literacy" [Tiab] OR "digital Health Literacy"[Tiab] OR “Scoping review”[Title] OR “Systematic Review” [Title] OR “Child*”[Tiab]	1,510
#9	#7 NOT #8	927

Database: **Web of Science**
Search date: 13/02/2024

	**Search strategy**	**Results**
#1	ALL=Exercise OR ALL="Sedentary Behavior"	718,572
#2	(TI="Physical activ*" OR AB="Physical activ*") OR (TI="Physical inactiv*" OR AB="Physical inactiv*") OR (TI=Exercis* OR AB=Exercis*) OR (TI="Sedentary Behav*" OR AB="Sedentary Behav*")	617,853
#3	#1 OR #2	824,098
#4	(ALL="Health Literacy")	20,482
#5	(TI="Health numeracy" OR AB="Health numeracy") OR (TI="Health Knowledge" OR AB="Health Knowledge") OR (TI="Health Literacy" OR AB="Health Literacy")	19,679
#6	#4 OR #5	31,886
#7	#3 AND #6	1,896
#8	(TI="Digital literacy" OR AB="Digital literacy") OR (TI=eliteracy OR AB=eliteracy) OR (TI="physical literacy" OR AB="physical literacy") OR (TI="digital Health Literacy" OR AB="digital Health Literacy") OR TI="Scoping review" OR TI="Systematic Review" OR TI=Child*	1,329,923
#9	#7 NOT #8	1,205

Database: APA PsycInfo
Search date: 13/02/2024

	Search strategy	Results
#1	MH Exercise+ OR MH "Sedentary Behavior+" OR TI "Physical activ*" OR AB "Physical activ*" OR TI "Physical inactiv*" OR AB "Physical inactiv*" OR TI Exercis* OR AB Exercis* TI "Sedentary Behav*" OR AB "Sedentary Behav*"	62,553
#2	MH "Health Literacy+" OR TI "Health numeracy" OR AB "Health numeracy" OR TI "Health Knowledge" OR AB "Health Knowledge" OR TI “Health Literacy” OR AB “Health Literacy”	6,943
#3	#1 AND # 2	232
#4	TI "Digital literacy" OR AB "Digital literacy" OR TI eliteracy OR AB eliteracy OR TI "physical literacy" OR AB "physical literacy" OR TI "digital Health Literacy" OR AB "digital Health Literacy" OR TI "Scoping review" OR TI "Systematic Review" OR Child*	1,037,604
#5	#3 NOT #4	186

Database: Medline
Search date: 13/02/2024

	Search strategy	Results
#1	MHX=(Exercise OR "Sedentary Behavior")	254,289
#2	(TI=("Physical activ*" OR "Physical inactiv*" OR Exercis* OR "Sedentary Behav*") OR AB=("Physical activ*" OR "Physical inactiv*" OR Exercis* OR "Sedentary Behav*"))	201,285
#3	#1 OR #2	582,123
#4	MHX=(“Health Literacy”)	9,666
#5	((TI=("Health Literacy" OR "Health numeracy" OR "Health numeracy" OR "Health Knowledge" OR)) OR AB=("Health Literacy" OR "Health numeracy" OR "Health numeracy" OR "Health Knowledge" OR))	18,210
#6	#4 OR #5	58,610
#7	#3 AND #6	3,843
#8	(TI=("Digital literacy" OR eliteracy OR "physical literacy" OR "digital Health Literacy" OR “Scoping review” OR “Systematic Review” OR “Child*”) OR AB=("Digital literacy" OR eliteracy OR "physical literacy" OR "digital Health Literacy" OR “Scoping review” OR “Systematic Review” OR “Child*”))	1,951,253
#9	#7 NOT #8	700

Database: **SCOPUS**
Search date: 13/02/2024

	Search strategy	Results
#1	(INDEXTERMS(Exercise)) OR (TITLE-ABS-KEY({Physical activ*} OR {Physical inactiv*} OR exercis* OR {Sedentary Behav*})) AND (TITLE-ABS-KEY({Health Literacy} OR {Health numeracy} OR {Health Knowledge})) OR	646

	(INDEXTERMS("Health Literacy")) AND NOT (TITLE-ABS-KEY ({Digital literacy} OR eliteracy OR {physical literacy} OR {Digital Health Literacy} OR {Scoping review} OR {Systematic Review} OR child*)) AND (LIMIT-TO (LANGUAGE,"English")) AND (EXCLUDE (EXACTKEYWORD,"Health Knowledge, Attitudes, Practice")) AND (EXCLUDE (SUBJAREA,"BIOC") OR EXCLUDE (SUBJAREA,"ENVI") OR EXCLUDE (SUBJAREA,"COMP") OR EXCLUDE (SUBJAREA,"PHAR") OR EXCLUDE (SUBJAREA,"EART") OR EXCLUDE (SUBJAREA,"ECON") OR EXCLUDE (SUBJAREA,"CENG") OR EXCLUDE (SUBJAREA,"DENT") OR EXCLUDE (SUBJAREA,"MATH") OR EXCLUDE (SUBJAREA,"BUSI") OR EXCLUDE (SUBJAREA,"IMMU") OR EXCLUDE (SUBJAREA,"NEUR") OR EXCLUDE (SUBJAREA,"ARTS") OR EXCLUDE (SUBJAREA,"AGRI") OR EXCLUDE (SUBJAREA,"ENGI"))	

Database: CINAHL Plus
Search date: 13/02/2024

	Search strategy	Results
	(((((MH Exercise+) OR (MH "Sedentary Behavior+")) OR ((TI "Physical activ*" OR AB "Physical activ*") OR (TI "Physical inactiv*" OR AB "Physical inactiv*") OR (TI Exercis* OR AB Exercis*) OR (TI "Sedentary Behav*" OR AB "Sedentary Behav*"))) AND (((MH "Health Literacy+")) OR ((TI "Health numeracy" OR AB "Health numeracy") OR (TI "Health Knowledge" OR AB "Health Knowledge") OR (TI "Health Literacy" OR AB "Health Literacy")))) NOT ((TI "Digital literacy" OR AB "Digital literacy") OR (TI eliteracy OR AB eliteracy) OR (TI "physical literacy" OR AB "physical literacy") OR (TI "digital Health Literacy" OR AB "digital Health Literacy") OR (TI "Scoping review") OR (TI "Systematic Review") OR Child*))	508
